# Cross-lineage 5-methylcytosine methylome profiling reveals methylated divergence among *Toxoplasma gondii* tachyzoites of the three major clonal lineages

**DOI:** 10.1186/s40249-025-01358-w

**Published:** 2025-08-19

**Authors:** Xiao-Nan Zheng, Hong-Yu Song, Hany M. Elsheikha, Chen-Ran Tian, Xing Tian, Qing Liu, Wen-Bin Zheng, Xing-Quan Zhu

**Affiliations:** 1 Laboratory of Parasitic Diseases, College of Veterinary Medicine, Shanxi Agricultural University, Taigu, 030801 Shanxi Province People’s Republic of China; 2 Faculty of Medicine and Health Sciences, School of Veterinary Medicine and Science, University of Nottingham, Sutton Bonington Campus, Loughborough, LE12 5RD UK

**Keywords:** *Toxoplasma gondii*, Toxoplasmosis, 5-methylcytosine, Methylated RNA immunoprecipitation sequencing, RNA sequencing, Epitranscriptomic regulation

## Abstract

**Background:**

*Toxoplasma gondii* is a globally widespread zoonotic parasite, infecting nearly one-third of the human population, often leading to chronic, latent infections. Among the emerging layers of gene regulation, 5-methylcytosine (m^5^C) has emerged as a pivotal post-transcriptional modification in eukaryotes. Despite its growing recognition in various species, the epitranscriptomic landscape of m^5^C in the tachyzoite stage of *T. gondii* remains largely unexplored. To address this gap, we performed the first comprehensive m^5^C methylation profiling across three major *T. gondii* genotypes—RH (type I), ME49 (type II), and VEG (type III).

**Methods:**

The comparative m^5^C methylation analysis was carried out using methylated RNA immunoprecipitation sequencing (MeRIP-Seq) combined with RNA sequencing (RNA-Seq). Differentially m^5^C-methylated genes (DMMGs) were functionally annotated via Gene Ontology (GO) and Kyoto Encyclopedia of Genes and Genomes (KEGG) pathway enrichment analyses. By combining methylation and transcriptomic data, we uncovered strain-specific correlations between m^5^C modifications and gene expression. Additionally, expression and methylation patterns of potential regulators identified via BLASTP searches were examined. Statistical analyses were determined by one-way ANOVA.

**Results:**

Our analysis revealed a total of 5129, 4968, and 4577 m^5^C-methylated genes in RH, ME49, and VEG tachyzoites, respectively, with methylation predominantly enriched in the coding sequences. Comparative analysis across different strains uncovered 1710, 1131, and 784 DMMGs in RH versus ME49, RH versus VEG, and ME49 versus VEG, respectively. Functional enrichment analysis highlighted key biological processes, including catalytic activity, transport, phospholipid metabolism and transcription regulation. Furthermore, KEGG pathway analysis identified critical m^5^C-regulated processes such as nucleocytoplasmic transport, DNA replication, and ATP-dependent chromatin remodeling. Virulence-associated secretory effectors exhibited hypermethylation in more virulent strains, such as GRA39 and ROP35. Additionally, several putative m^5^C regulators displayed genotype-specific or conserved expression and methylation patterns.

**Conclusions:**

This study presents the first m^5^C epitranscriptomic atlas of *T. gondii* tachyzoites, revealing both conserved and genotype-specific mRNA modification networks. These insights significantly increased the understanding of the regulatory role of m^5^C in *T. gondii* pathogenesis and open promising avenues for the development of vaccines and therapeutics aimed at combating zoonotic toxoplasmosis.

**Graphical Abstract:**

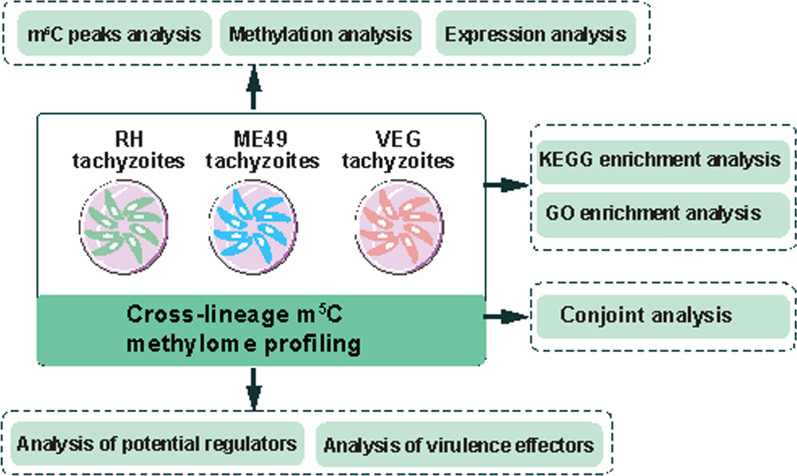

**Supplementary Information:**

The online version contains supplementary material available at 10.1186/s40249-025-01358-w.

## Background

*Toxoplasma gondii* is a zoonotic protozoan parasite infecting almost all warm-blooded animals and humans [1]. It is estimated that nearly one-third of the global population harbors chronic *T. gondii* infections, primarily acquired through the ingestion of contaminated water or food containing tissue cysts or oocysts [2]. While most infections remain asymptomatic, immunocompromised individuals face a heightened risk of life-threatening toxoplasmosis due to the reactivation and proliferation of tachyzoites from latent tissue cysts [3]. Furthermore, accumulating evidence suggests a potential link between chronic *T. gondii* infection and the pathogenesis of neuropsychiatric disorders in humans, including schizophrenia and other mental health conditions [2–4]. Beyond its impact on human health, *T. gondii* is a major etiological agent of pregnancy loss and congenital abnormalities in livestock species such as sheep, goats, and swine, causing substantial economic losses to the global livestock industry [1].

Genetic characterization of *T. gondii* isolates from wildlife using restriction fragment length polymorphism (RFLP) analysis has identified three predominant clonal lineages—types I, II, and III—each exhibiting distinct virulence profiles in murine models [5]. Type I strains, such as RH, are highly virulent, causing 100% mortality in mice with as few as one tachyzoite [5–7]. In contrast, type II (ME49) strain shows intermediate virulence depending on mouse strain, while type III (VEG) strains are considered avirulent [5–7]. These genotypes also display distinct geographic distributions, with types I–III prevalent in North America and Europe, and more genetically diverse, non-archetypal strains dominating in South America, Asia, and Africa [5, 8, 9]. Among these, type II strains are most frequently associated with human toxoplasmosis cases [10]. The RH (type I), ME49 (type II), and VEG (type III) strains serve as key reference models for experimental studies. However, the molecular mechanisms driving their distinct virulence remain incompletely understood, highlighting the need for further exploration of their genetic and epitranscriptomic features.

RNA modifications constitute a critical regulatory layer in gene expression and translation, influencing processes such as RNA stability, processing, macromolecular interactions, and translation efficiency [11–13]. These modifications occur across diverse RNA species, including messenger RNA (mRNAs), transfer RNAs (tRNAs), ribosomal RNAs (rRNAs), and non-coding RNAs (ncRNAs) [11, 14–16]. To date, more than 100 distinct RNA modifications have been identified, with key modifications such as N6-methyladenosine (m^6^A), 5-methylcytosine (m^5^C), N1-methyladenosine (m^1^A), and N7-methylguanosine (m^7^G) playing fundamental roles in cellular regulation [11, 12, 17]. Emerging research highlights the importance of dynamic RNA modifications in parasite pathogenesis, affecting virulence, replication, developmental transitions, and host–pathogen interactions [11].

Among these, m^5^C, characterized by methylation at the fifth carbon position of cytosine, has been observed across diverse RNA species, including mRNA, rRNA, tRNA and ncRNAs [14, 18]. In mRNA, m^5^C modifications are differentially distributed in the coding sequence (CDS), 3′ untranslated region (3′UTR), and 5′UTR, with distinct functional consequences on gene expression in various biological processes across species [14]. For instance, m^5^C modification within CDS of mRNA stabilizes spermine oxidase mRNA in esophageal squamous cell carcinoma [19], while enhancing the translation efficiency of quiescin sulfhydryl oxidase 1 in non-small-cell lung cancer [20]. m^5^C modification in the 5′UTR of nuclear factor erythroid 2-related factor 2 upregulates its expression by enhancing mRNA stability rather than influencing its translation [21]. Modification in the 3′UTR stabilizes mRNA [22–24], and promotes protein translation level by influencing translation termination or interacting with RNA binding proteins [25–27]. In tRNAs, the modification stabilizes the secondary structure, impacting translation accuracy and efficiency [14].

The dynamic regulation of m^5^C involves a complex interplay of catalytic enzymes, including “writers” (methyltransferases) depositing the modification, “readers” (methylation-binding proteins) recognizing it, and “erasers” (demethylases) removing it [11, 14, 15]. This regulatory interplay governs critical molecular functions such as mRNA export, RNA stability, translation, and long-distance RNA transport [14]. In eukaryotes, the m^5^C methyltransferase NOL1/NOP2/SUN domain family member 2 (NSUN2) and the reader protein Y-box binding protein 1 (YBX1) have been implicated in oncogenic transcriptional regulation [18, 28–30]. Moreover, NSUN1 has been shown to play a role in HIV-1 transcription and replication [31]. In protozoan parasites, m^5^C regulatory mechanisms also control essential developmental processes. For instance, *Plasmodium falciparum* employs PfNSUN2 for mRNA stability and PfDNMT2 for tRNA cytosine methylation, both of which are crucial for sexual differentiation [32, 33]. Additionally, m^5^C modification is involved in regulating the stress response, mitochondrial activity, nerve and brain development, gametogenesis, and embryogenesis [14]. Given these fundamental roles of m^5^C in cellular regulation, this modification could also play a pivotal role in *T. gondii* pathogenesis, particularly in processes such as tachyzoite proliferation, immune evasion, differentiation and the maintenance of latent infection.

Despite these advances, a systematic characterization of m^5^C modification patterns in *T. gondii* remains unexplored, particularly with respect to lineage-specific epitranscriptomic variations. To address this gap, we performed comparative m^5^C profiling of intracellular tachyzoites from the RH, ME49, and VEG reference strains using methylated RNA immunoprecipitation sequencing (MeRIP-Seq), alongside transcriptome profiling. This study not only expands our understanding of the epitranscriptomic landscape in *T. gondii*, but also provides valuable insights into conserved and strain-specific RNA modification pathways, which may serve as potential targets for anti-*Toxoplasma* interventions.

## Methods

### *T. gondii* strains and cell culture

We used three *T. gondii* strains: RH (type I), ME49 (type II), and VEG (type III). These strains were maintained in confluent monolayer of human foreskin fibroblast (HFF) cells (ATCC SCRC-1041), as previously described [34]. HFFs were cultured in Dulbecco’s Modified Eagle Medium (DMEM, Gibco, Suzhou, China) supplemented with 10% fetal bovine serum (FBS, Gibco, Australia), 10 mmol/L HEPES (pH 7.2, Solarbio, Beijing, China), and penicillin–streptomycin solution (100 U/ml penicillin, 100 μg/ml streptomycin, Solarbio, Beijing, China). The cells were maintained in a humidified incubator at 37 °C with 5% CO_2_ [34–36]. For infection, HFF monolayers were infected with tachyzoites and incubated for 40 h. After the incubation period, the cells were washed twice with cold PBS, then scraped and centrifuged at 2500 × *g* for 10 min. The resulting cell pellets were frozen in liquid nitrogen for subsequent analysis. Three biological replicates per *T. gondii* genotype were processed for subsequent methylome and transcriptome profiling, as previously described [37].

### m^5^C-MeRIP Sequencing (m^5^C-MeRIP-Seq) and RNA-Seq

**Fig. 1:**
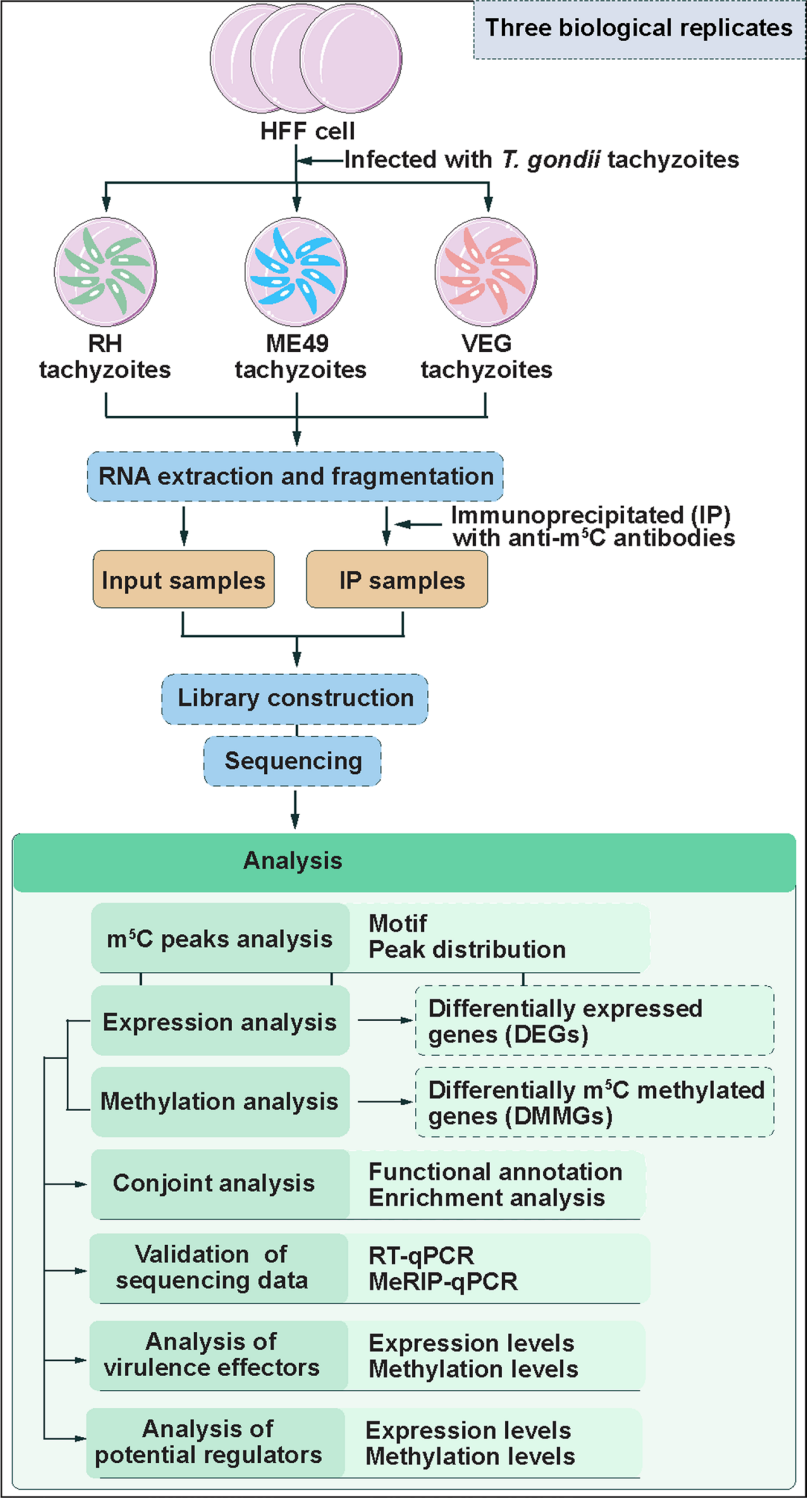
Schematic overview of the study workflow from data collection to analysis. *HFF* human foreskin fibroblast cells, *RH Toxoplasma* wild-type I strain, *ME49 Toxoplasma* wild-type II strain, *VEG Toxoplasma* wild-type III strain, *m*^*5*^*C* 5-methylcytosine, *IP* immunoprecipitation, *RT-qPCR* quantitative reverse transcription PCR, *MeRIP-qPCR* methylated RNA immunoprecipitation-qPCR

As depicted in the experimental workflow (Fig. 1), total RNA was extracted from *T. gondii* tachyzoites of the RH, ME49, and VEG genotypes using TRIzol^®^ Reagent (Invitrogen, Carilsbad, CA, USA). RNA concentration was quantified with a NanoDrop^™^ (ND-1000, Thermo Fisher Scientific, Waltham, MA, USA), and RNA integrity was assessed through denaturing agarose gel electrophoresis. Ribosomal RNA (rRNA) was then removed using the GenSeq^®^ rRNA Removal Kit (GenSeq Inc., Shanghai, China) [38]. The RNA samples were divided into two aliquots: one was used as the input RNA control for transcriptome profiling, and the other was subjected to m^5^C methylated RNA immunoprecipitation sequencing (m^5^C-MeRIP-Seq). Both sequencing workflows were conducted by Cloudseq Inc (Shanghai, China). For m^5^C-MeRIP-Seq, immunoprecipitation (IP) was performed using the GenSeq m^5^C MeRIP Kit (GenSeq Inc., Shanghai, China) [39]. Briefly, rRNA-depleted samples were fragmented to ~ 200 nt using RNA fragmentation reagents, then incubated with protein A/G beads conjugated to anti-m^5^C antibodies (Abcam, Cambridge, UK) for 4 h at 4 ℃ with rotation. After incubation, the antibody-RNA complexes were sequentially washed and then eluted in nuclease-free water. RNA from both the IP and input groups was then converted into sequencing libraries using the GenSeq^®^ Low Input Whole RNA Library Prep Kit (GenSeq Inc., Shanghai, China). Library quality was evaluated with an Agilent 2100 Bioanalyzer (Agilent, California, USA), and the libraries were sequenced on an Illumina platform (Illumina, San Diego, CA, USA). Both m^5^C-MeRIP-Seq and RNA-Seq experiments were conducted in three biological replicates.

### Identification of differentially methylated peaks and genes

Raw sequencing reads were first quality-checked using a Q30 threshold. Low-quality reads were removed with Cutadapt R 1.18 (Stockholm University, Stockholm, Sweden) [40], and the remaining high-quality reads were aligned to the *T. gondii* ME49 reference genome (ToxoDB; https://toxodb.org) using HISAT2 R 2.2.1 (Johns Hopkins University, Baltimore, USA) [41]. Since host RNA was not depleted prior to sequencing, the total RNA included both parasite- and host-derived transcripts. To minimize host RNA interference, only reads that uniquely mapped to the *T. gondii* genome were retained for downstream analyses. Methylation-enriched regions (i.e., methylation peaks) within mRNA were identified using MACS R 1.4.2 (Broad Institute of Massachusetts Institute of Technology and Harvard, Cambridge, USA) applying a significance threshold of *P* value ≤ 10⁻^5^ and a fold enrichment ≥ 2. IP samples were treated as the experimental groups, while matched input samples served as controls [42]. Differential methylation between groups were assessed using diffReps R 1.55.6 (Icahn School of Medicine at Mount Sinai, New York, USA), with the same significance thresholds [43]. Motif analysis was performed using DREME R 5.3.0 (University of Queensland & University of Washington, Brisbane, Australia/Seattle, USA).

### Identification of differentially expressed genes and functional analysis of differentially m^5^C-methylated genes (DMMGs)

Transcriptomic profiling was performed using edgeR package R 4.2.1 (Walter and Eliza Hall Institute of Medical Research, Melbourne, Australia) to identify differentially expressed genes (DEGs) with a *Q* value (adjusted *P* value) ≤ 0.05, absolute log_2_ fold change (FC) ≥ 1 and uniquely mapped reads to the *T. gondii* genome. Functional annotation of differentially methylated m^5^C genes (DMMGs) was conducted using the topGO package R. 2.42.0 (Swiss Federal Institute of Technology Zurich, Zurich, Switzerland), which revealed significant Gene Ontology (GO) enrichment (*P* ≤ 0.05). The top five terms from each GO category were visualized. Kyoto Encyclopedia of Genes and Genomes (KEGG) pathway enrichments analysis of DMMGs was performed using the KEGG database (https://genome.jp/kegg/) [44], with pathways exhibiting a *P* value ≤ 0.05 considered statistically significant.

### Validation by quantitative reverse transcription PCR (RT-qPCR) and MeRIP-qPCR

To validate the multi-omics results from m^5^C-MeRIP-Seq and RNA-Seq analysis, RT-qPCR and MeRIP-qPCR assays were performed on both input and IP samples. RT-qPCR was conducted using a QuantStudio 5 Real-Time PCR System (Thermo Fisher Scientific, Waltham, MA, USA), as described previously [38]. Actin (TGME49_209030) was used as the endogenous normalization control. The relative expression of DEGs was quantified using the comparative cycle threshold (Ct) method (2^−ΔΔCt^), where the first ΔCt represents the difference in Ct values between the target gene and the reference (housekeeping) gene, and the second ΔΔCt represents the difference of ΔCt between two compared *T. gondii* strains. m^5^C methylation levels in IP samples were calculated relative to input baselines using the following formula: %(IP/Input) = 2^[Ct(input)−Ct(IP)]^ × DF × 100, where DF denotes dilution factor [38]. Primer sequences for validation are provided in Additional file 1: Table S1 and Additional file 2: Table S2.

### Data analysis

All experiments were conducted in independent biological triplicate with continuous variable data presented as mean ± standard deviation (SD). Differentially methylated mRNA sites were identified using diffReps with stringent thresholds (*P* value ≤ 10⁻^5^ and fold enrichment ≥ 2). Inter-group differences in expression and methylation validation experiments were assessed by one-way Analysis of Variance (ANOVA) using GraphPad Prism R 10.0 (GraphPad Software, San Diego, USA), with statistical significance set at *P* ≤ 0.05. Significance levels are denotes as ****P ≤ 0.0001, ***P ≤ 0.001, **P ≤ 0.01, *P ≤ 0.05 and NS (not significant) for *P* > 0.05.

## Results

### Lineage-specific m^5^C methylation patterns in *T. gondii* tachyzoite mRNA

**Fig. 2:**
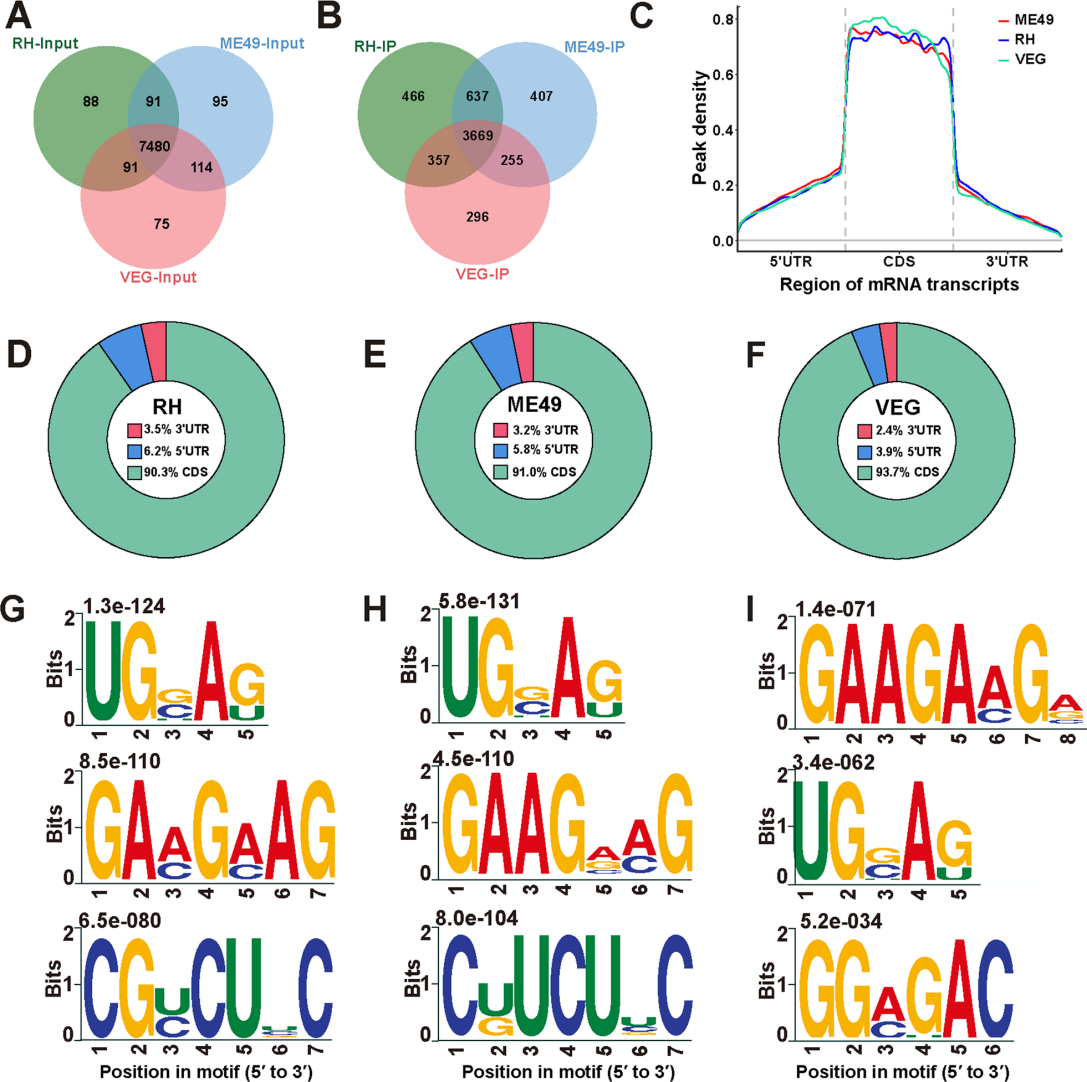
Transcriptome-wide m^5^C modification landscape in *Toxoplasma gondii* tachyzoites of RH, ME49, and VEG strains. **A** Venn diagram showing the number of shared and unique expressed genes across strains. **B** Venn diagram illustrating the distribution of genes with m^5^C methylation peaks. **C** Metagene plots depicting the distribution of m^5^C peaks across mRNA transcripts. **D**–**F** Pie charts showing the proportion of m^5^C peaks located in the coding sequence (CDS), 3′ untranslated region (3′UTR), and 5′UTR for the RH, ME49, and VEG strains, respectively. **G**–**I** Top three sequence motifs enriched in the mRNAs of the RH, ME49, and VEG strains, respectively. *RH Toxoplasma* wild-type I strain, *ME49 Toxoplasma* wild-type II strain, *VEG Toxoplasma* wild-type III strain, *IP* immunoprecipitation

A total of 8034 genes were identified in *T. gondii* tachyzoites, with 7480 genes conserved across all lineages. Of these, 88, 95, and 75 genes were uniquely present in RH, ME49, and VEG strains, respectively (Fig. 2A). m^5^C-MeRIP analysis revealed m^5^C methylation peaks in 6087 genes, of which 3669 genes were consistently methylated across all tachyzoite lineages (Fig. 2B). Further comparative analysis identified 637, 357, and 255 methylated genes shared between the RH-ME49, RH-VEG, and ME49-VEG strain pairs, respectively. Additionally, 466, 407, and 296 genes exhibited lineage-specific methylation in the RH, ME49, and VEG strains (Fig. 2B). Chromosomal mapping revealed a predominant m^5^C deposition on the X chromosome across all *T. gondii* tachyzoites (Additional file 3: Figure S1A–C). Analysis of m^5^C peak distribution across genes showed that most genes harbored 1–3 m^5^C peaks (Additional file 3: Figure S1D–F). Importantly, methylation patterns exhibited striking conservation, with the highest enrichment observed in the CDS region (Fig. 2C). Specifically, in RH tachyzoites, 90.3% of m^5^C peaks were located within the CDS, while 3.5% and 6.2% were found in the 3′UTR and 5′UTR, respectively (Fig. 2D). This pattern was consistent across the ME49 and VEG strains (Fig. 2E, F). Additionally, multiple m^5^C sequence motifs were identified, with the top three motifs for each lineage of *T. gondii* tachyzoites illustrated in Fig. 2G–I.

### Identification of DMMGs

**Fig. 3:**
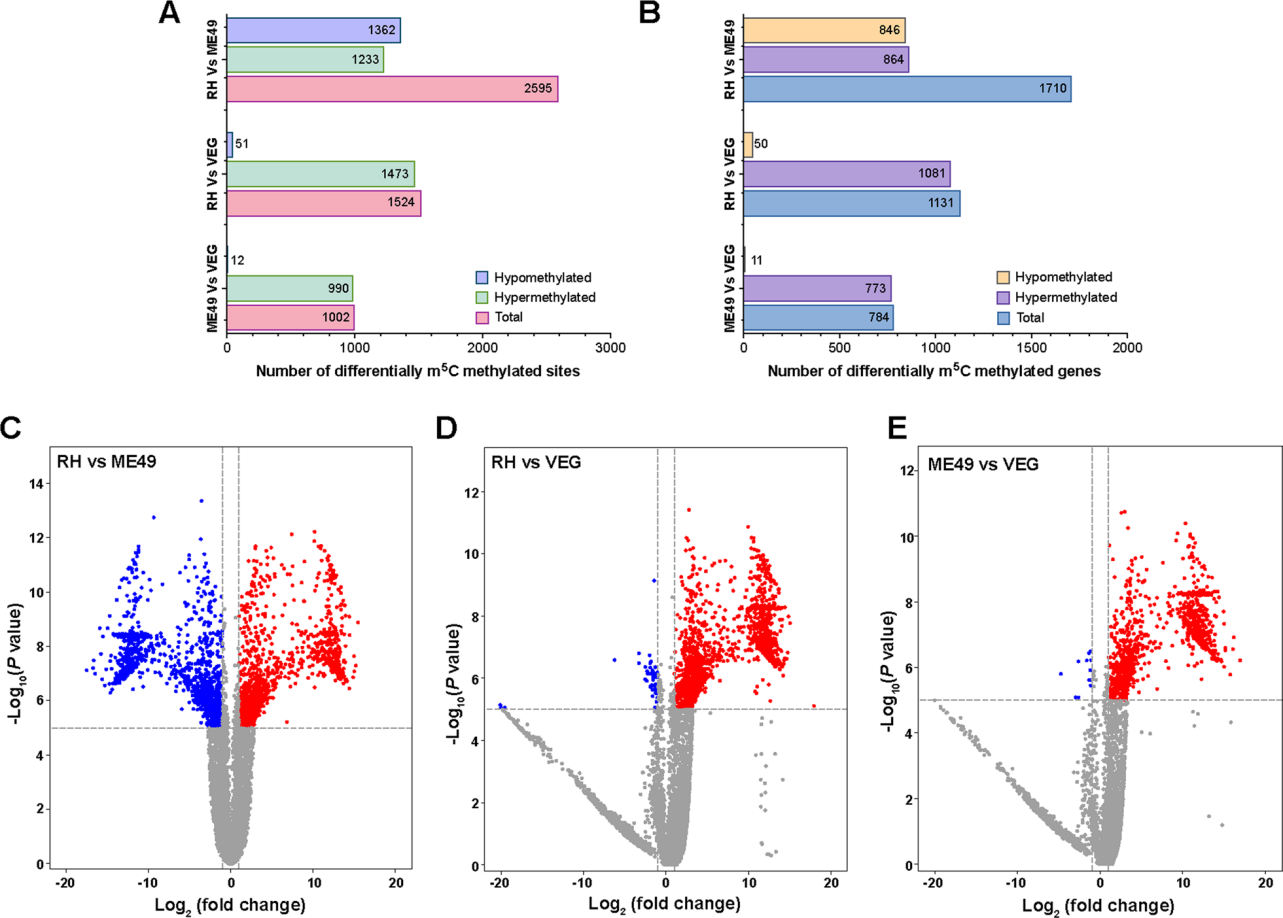
Differential m^5^C methylation profiles in *Toxoplasma gondii* tachyzoites across the RH, ME49, and VEG strains. **A** Histogram showing the number of differentially methylated sites between strain comparisons. **B** Histogram illustrating the number of genes with differentially methylated peaks. **C**–**E** Volcano plots displaying differentially methylated m^5^C peaks in the RH versus ME49, RH versus VEG, and ME49 versus VEG comparisons, respectively. Differentially methylated m^5^C peaks are highlighted, with hypermethylated sites in red, hypomethylated sites in blue and sites without significant methylation in grey. *RH Toxoplasma* wild-type I strain, *ME49 Toxoplasma* wild-type II strain, *VEG Toxoplasma* wild-type III strain

Comparative methylome analysis identified 2595 differentially methylated m^5^C peaks across 1710 genes in the RH versus ME49 tachyzoite comparison. Of these, 1362 peaks were hypomethylated in 846 genes, while 1233 peaks were hypermethylated in 864 genes in RH relative to ME49 (Fig. 3A–C; Additional file 4: Table S3). In the RH versus VEG comparison, 1524 differential m^5^C methylation peaks were detected in 1131 genes, with a marked predominance of hypermethylation—1473 peaks—compared to only 51 hypomethylated peaks (Fig. 3A, B, D; Additional file 4: Table S3). Similarly, the ME49 versus VEG comparison revealed a clear hypermethylation bias, with 990 significantly hypermethylated peaks in 773 genes, while only 12 peaks were hypomethylated in 11 genes (Fig. 3A, B, E; Additional file 4: Table S3). Chromosomal mapping revealed a conserved pattern of differential m^5^C peak distribution across all comparisons (Additional file 3: Figure S2, A–C). In all comparisons, DMMGs containing 1–3 m^5^C peaks were the most prevalent (Additional file 3: Figure S2, D–F).

### Functional GO enrichment analysis of DMMGs

**Fig. 4:**
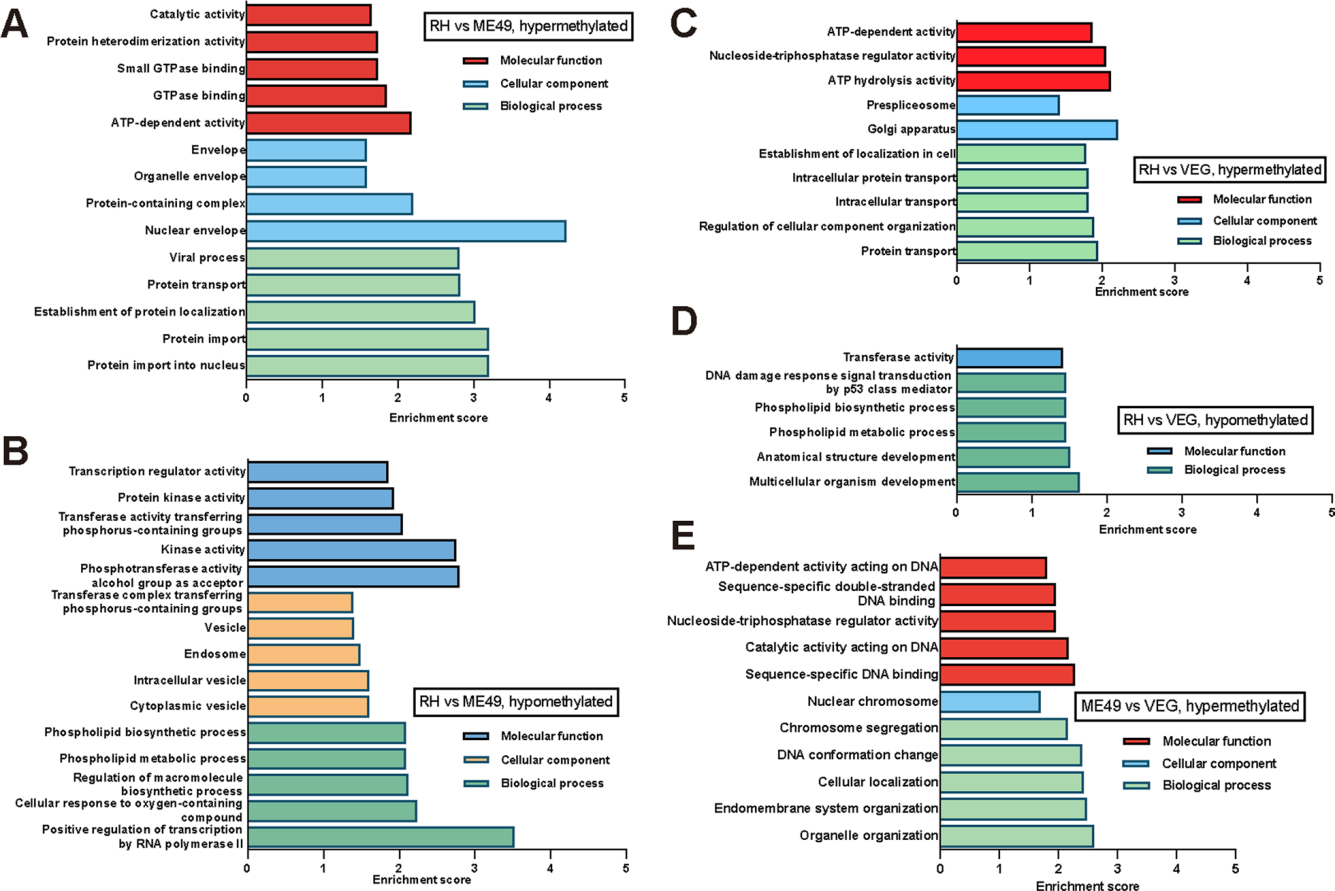
Gene Ontology (GO) enrichment analysis of differentially methylated genes. **A**, **B** GO enrichment analysis of genes with hypermethylated and hypomethylated peaks in the RH versus ME49 comparison. **C**, **D** GO enrichment analysis of hypermethylated and hypomethylated genes in the RH versus VEG comparison. **E** GO enrichment of hypermethylated genes in ME49 versus VEG. *RH Toxoplasma* wild-type I strain, *ME49 Toxoplasma* wild-type II strain, *VEG Toxoplasma* wild-type III strain

GO enrichment analysis was performed to annotate the functional roles of DMMGs, revealing distinct epigenetic regulatory patterns across the strain comparisons. In the RH versus ME49 comparison, hypermethylated genes were predominantly enriched in molecular functions (MF) related to energy metabolism, including ATP-dependent activity, GTPase, and small GTPase binding. Cellular components (CC) were mainly localized to the nuclear envelope and protein complexes, while biological processes (BP) were involved in protein trafficking, such as protein import into the nucleus and the establishment of protein localization (Fig. 4A; Additional file 5: Table S4). In contrast, hypomethylated genes in RH versus ME49 exhibited a clear focus on transcriptional regulation, particularly in RNA polymerase II-mediated transcription regulation and phospholipid metabolism. These processes included positive regulation of transcription, cellular response to oxygen-containing compounds, macromolecule biosynthesis regulation, and phospholipid biosynthesis (Fig. 4B; Additional file 5: Table S4). Cellular component analysis revealed that hypomethylated genes were predominantly localized to intracellular vesicular compartments (Fig. 4B; Additional file 5: Table S4). Molecular function annotations indicated a dual role of these genes in transcriptional regulation and catalytic activity (Fig. 4B; Additional file 5: Table S4).

In the RH versus VEG comparison, hypermethylated genes were enriched in molecular functions related to energy metabolism, including ATP-dependent activity, nucleoside-triphosphatase regulation, and ATP hydrolysis. These genes were primarily localized to RNA processing organelles (e.g., prespliceosome) and protein processing organelles (e.g., Golgi apparatus) within the cellular component category (Fig. 4C; Additional file 5: Table S4). Interestingly, protein trafficking pathways were similarly enriched in both RH versus VEG and RH versus ME49 comparisons (Fig. 4C; Additional file 5: Table S4). Conversely, hypomethylated genes in RH versus VEG were predominantly enriched in transferase activity, as well as developmental processes such as anatomical structure development and multicellular organism development. Additionally, these genes were involved in DNA damage response signaling, particularly through p53 class mediators (Fig. 4D; Additional file 5: Table S4). Phospholipid metabolic processes were also markedly enriched in hypomethylated genes, a trend shared with the hypomethylated profiles in the RH versus ME49 comparison (Fig. 4D; Additional file 5: Table S4).

In the ME49 versus VEG comparison, hypermethylation events pointed to DNA-centric regulatory mechanisms, with molecular functions enriched in sequence-specific DNA binding, ATP-dependent catalytic activity (Fig. 4E; Additional file 5: Table S4). Additionally, hypermethylated genes in this comparison were enriched in nuclear chromosome-related cellular components (Fig. 4E; Additional file 5: Table S4). The biological process enrichment for hypermethylated genes in ME49 versus VEG also aligned with organizational and localization processes observed in the other strain comparisons (Fig. 4E; Additional file 5: Table S4). The biological process enrichment for hypermethylated genes in ME49 versus VEG also aligned with organizational and localization processes observed in the other strain comparisons (Fig. 4E; Additional file 5: Table S4).

### KEGG pathway enrichment analysis of DMMGs

**Fig. 5:**
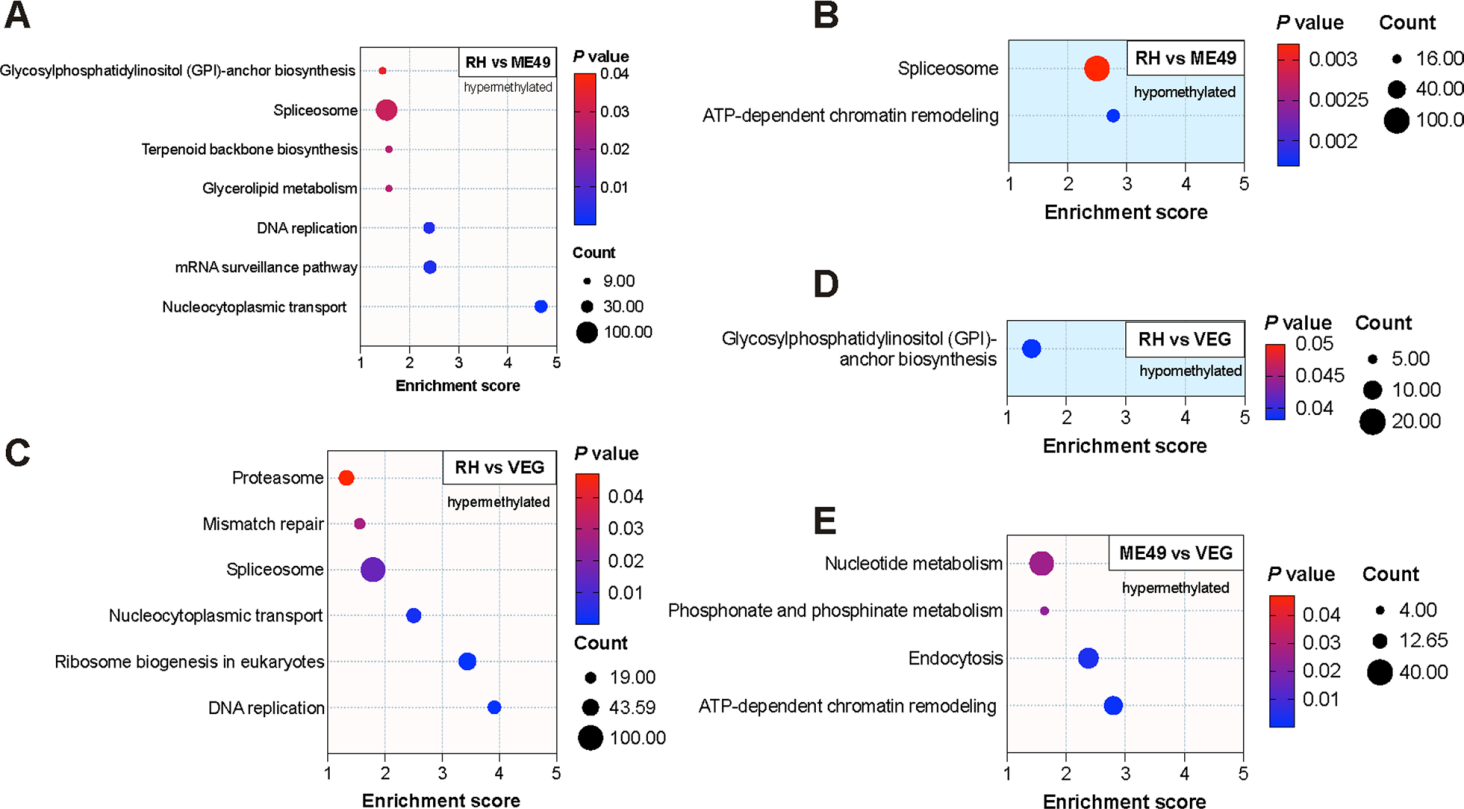
Kyoto Encyclopedia of Genes and Genomes (KEGG) pathway enrichment analysis of differentially methylated genes. **A**, **B** Pathway analysis of hypermethylated and hypomethylated genes in the RH versus ME49 comparison. **C**, **D** Pathway analysis of hypermethylated and hypomethylated genes in the RH versus VEG comparison. **E** KEGG enrichment of hypermethylated genes in the ME49 versus VEG comparison. *RH Toxoplasma* wild-type I strain, *ME49 Toxoplasma* wild-type II strain, *VEG Toxoplasma* wild-type III strain

KEGG pathway analysis of DMMGs revealed both conserved and strain-specific regulatory patterns. In the RH versus ME49 comparison, hypermethylated genes were predominantly enriched in pathways related to nucleocytoplasmic transport, mRNA surveillance, and DNA replication. In contrast, hypomethylated genes were associated with ATP-dependent chromatin remodeling (Fig. 5A, B; Additional file 6: Table S5). Interestingly, spliceosome pathway enrichment was observed in both hypermethylated and hypomethylated genes in this comparison (Fig. 5A, B; Additional file 6: Table S5). In the RH versus VEG comparison, hypermethylated genes were enriched in pathways associated with DNA replication, ribosome biogenesis in eukaryotes, and nucleocytoplasmic transport. Unique enrichment for hypomethylated genes was observed in the glycosylphosphatidylinositol (GPI)-anchor biosynthesis pathway (Fig. 5C, D; Additional file 6: Table S5). In the ME49 versus VEG comparison, hypermethylated genes were particularly enriched in pathways related to nucleotide and phosphonate metabolism, as well as endocytosis. The ATP-dependent chromatin remodeling pathway, which was enriched in hypomethylated genes in the RH versus ME49 analysis, was also observed in this comparison (Fig. 5E; Additional file 6: Table S5).

### Conjoint analysis of DMMGs and DEGs

**Fig. 6:**
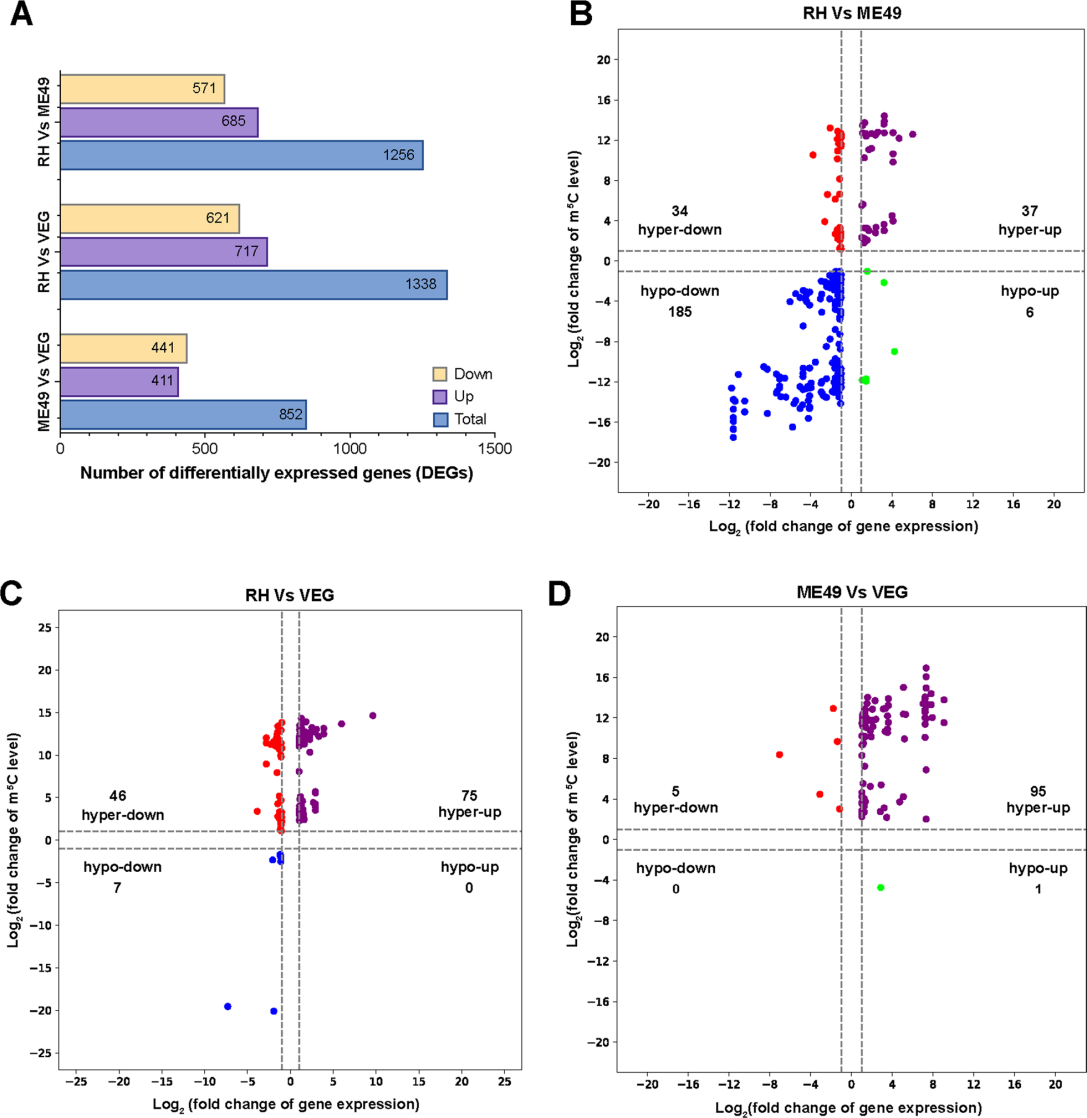
Differential gene expression and integrative analysis with m^5^C methylation in *T. gondii* tachyzoites. **A** Histogram showing the number of differentially expressed genes across the RH, ME49, and VEG strains. **B**–**D** Four-quadrant plots illustrating differentially methylated m^5^C peaks in conjunction with differentially expressed genes in the RH versus ME49, RH versus VEG, and ME49 versus VEG comparisons, respectively. *RH Toxoplasma* wild-type I strain, *ME49 Toxoplasma* wild-type II strain, *VEG Toxoplasma* wild-type III strain

RNA-Seq identified 1256 DEGs in the RH versus ME49 comparison, with 571 downregulated and 685 upregulated genes (Fig. 6A; Additional file 7: Table S6; Additional file 3: Figure S3A). The conjoint analysis of m^5^C-MeRIP and RNA-Seq data uncovered 262 peaks exhibiting concurrent changes in both methylation and expression. These were categorized as follows: 37 hypermethylated and upregulated (hyper-up) peaks, 34 hypermethylated and downregulated (hyper-down) peaks, 6 hypomethylated and upregulated (hypo-up) peaks, and 185 hypomethylated and downregulated (hypo-down) peaks (Fig. 6B; Additional file 8: Table S7). Remarkably, 43.0% of DEGs with differential methylation displayed multiple methylation peaks. This included three downregulated kinases (TGME49_266010, TGME49_288440, TGME49_218400) with more than six peaks, and three HECT-domain ubiquitin-transferases (upregulated TGME49_270580 and downregulated TGME49_280660, TGME49_209000) with over three differential methylation peaks (Additional file 8: Table S7).

In the RH versus VEG comparison, RNA-Seq analysis identified 1338 DEGs, with 621 downregulated and 717 upregulated genes (Fig. 6A; Additional file 7: Table S6; Additional file 3: Figure S3B). The conjoint analysis identified 75 hypermethylated and upregulated peaks (hyper-up), 46 hypermethylated and downregulated peaks (hyper-down), and 7 hypomethylated and downregulated peaks (hypo-down) (Fig. 6C; Additional file 8: Table S7). Interestingly, multi-peak methylation was observed in 31.0% of the methylation-associated DEGs. This included two hyper-up DEGs (TGME49_255290 and TGME49_244700) with five methylation peaks, and two downregulated genes (TGME49_280660 and TGME49_301400) with more than three methylation peaks (Additional file 8: Table S7).

In the ME49 versus VEG comparison, transcriptomic profiling identified 441 downregulated and 411 upregulated DEGs (Fig. 6A; Additional file 7: Table S6; Additional file 3: Figure S3C). Among the 852 DEGs, we identified 101 loci where methylation and expression co-varied, including 95 hypermethylated and upregulated peaks (hyper-up), 5 hypermethylated and downregulated peaks (hyper-down), and 1 hypomethylated and upregulated peak (hypo-up) (Fig. 6D; Additional file 8: Table S7). Interestingly, 28.8% of DEGs with significant methylation changes displayed multiple differential peaks. These included upregulated and hypermethylated genes such as the NEK kinase (TGME49_218400), phosphatidylinositol 3- and 4-kinase (TGME49_266010), and two hypothetical proteins (TGME49_225090 and TGME49_268220), all of which exhibited more than five differential peaks (Additional file 8: Table S7). Additionally, genes such as the apicomplexan monocarboxylate transporter 2 (AMT2, TGME49_297245), which plays a critical role in *T. gondii* virulence [45], as well as two major facilitator superfamily transporters (TGME49_268020 and TGME49_319740), exhibited a positive correlation between methylation and expression in the ME49 versus VEG comparison (Additional file 8: Table S7). The integrated analysis of m^5^C methylation and transcriptional profiles revealed significant associations between methylation changes and gene expression across *T. gondii* strains (Additional file 3: Figure S4).

**Fig. 7:**
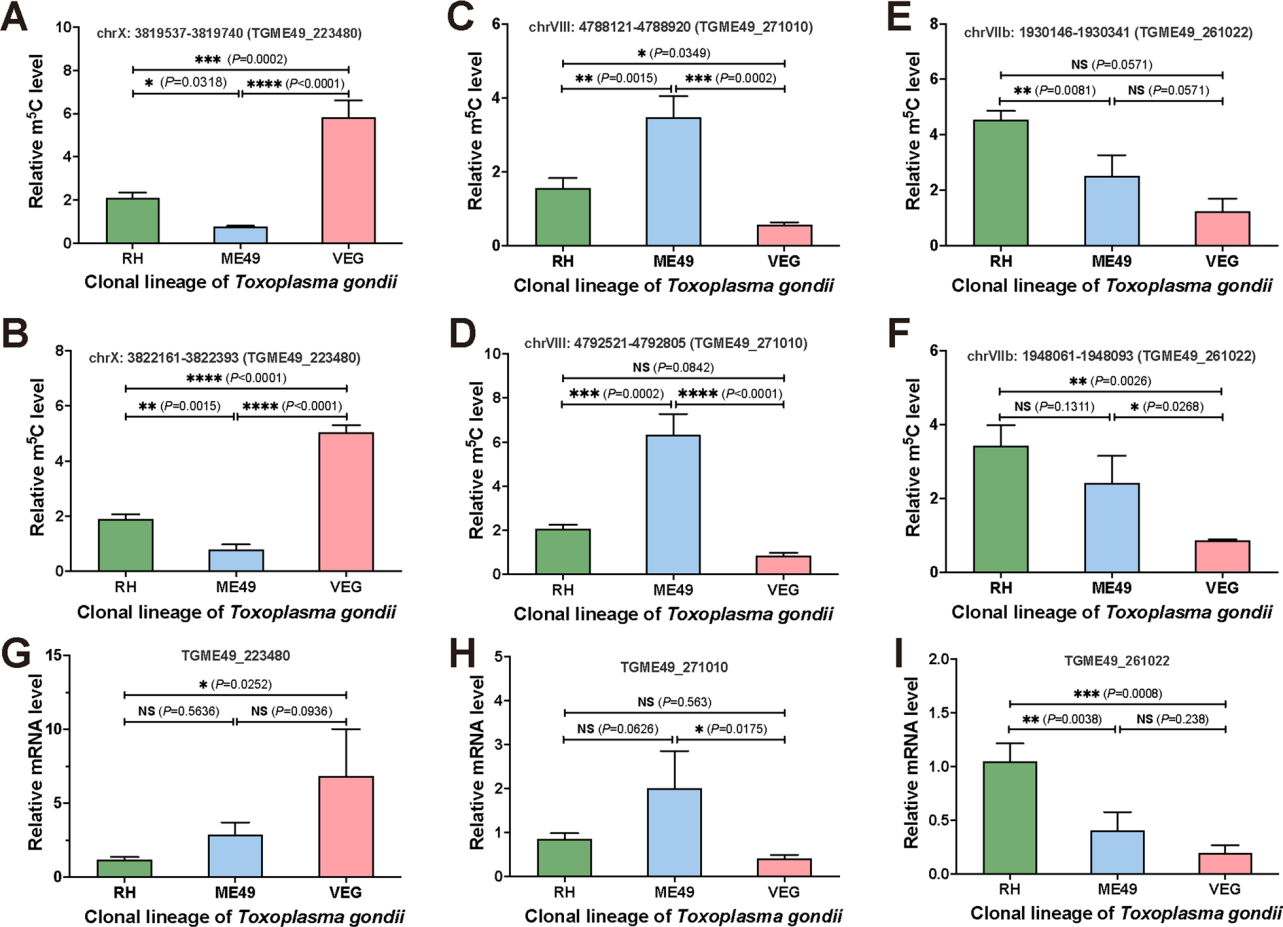
Validation of m^5^C methylation and gene expression levels. **A**–**F** MeRIP-qPCR validation of m^5^C methylation levels at two sites of *TGME49_223480*, *TGME49_271010*, and *TGME49_261022* across different *T. gondii* strains. **G**–**I** RT-qPCR validation of expression levels of *TGME49_223480*, *TGME49_271010*, and *TGME49_261022* in different *T. gondii* tachyzoite lineages, respectively. Statistical significance was tested by one-way analysis of variance (ANOVA). *RH Toxoplasma* wild-type I strain, *ME49 Toxoplasma* wild-type II strain, *VEG Toxoplasma* wild-type III strain, *MeRIP-qPCR* methylated RNA immunoprecipitation-quantitative reverse transcription PCR, *RT-qPCR* quantitative reverse transcription PCR

To validate the m^5^C-MeRIP data, we randomly selected two m^5^C sites from each of three candidate genes (TGME49_223480, TGME49_271010, and TGME49_261022) for methylation site verification using m^5^C-MeRIP-qPCR (Fig. 7). The results confirmed the differential methylation of these sites in mRNAs, with patterns that were consistent with the m^5^C-MeRIP data (Fig. 7A–F). Additionally, RT-qPCR analysis further corroborated the transcriptional profiles of these genes (Fig. 7G–I).

### m^5^C methylation and expression analysis of virulence-related effectors

**Fig. 8:**
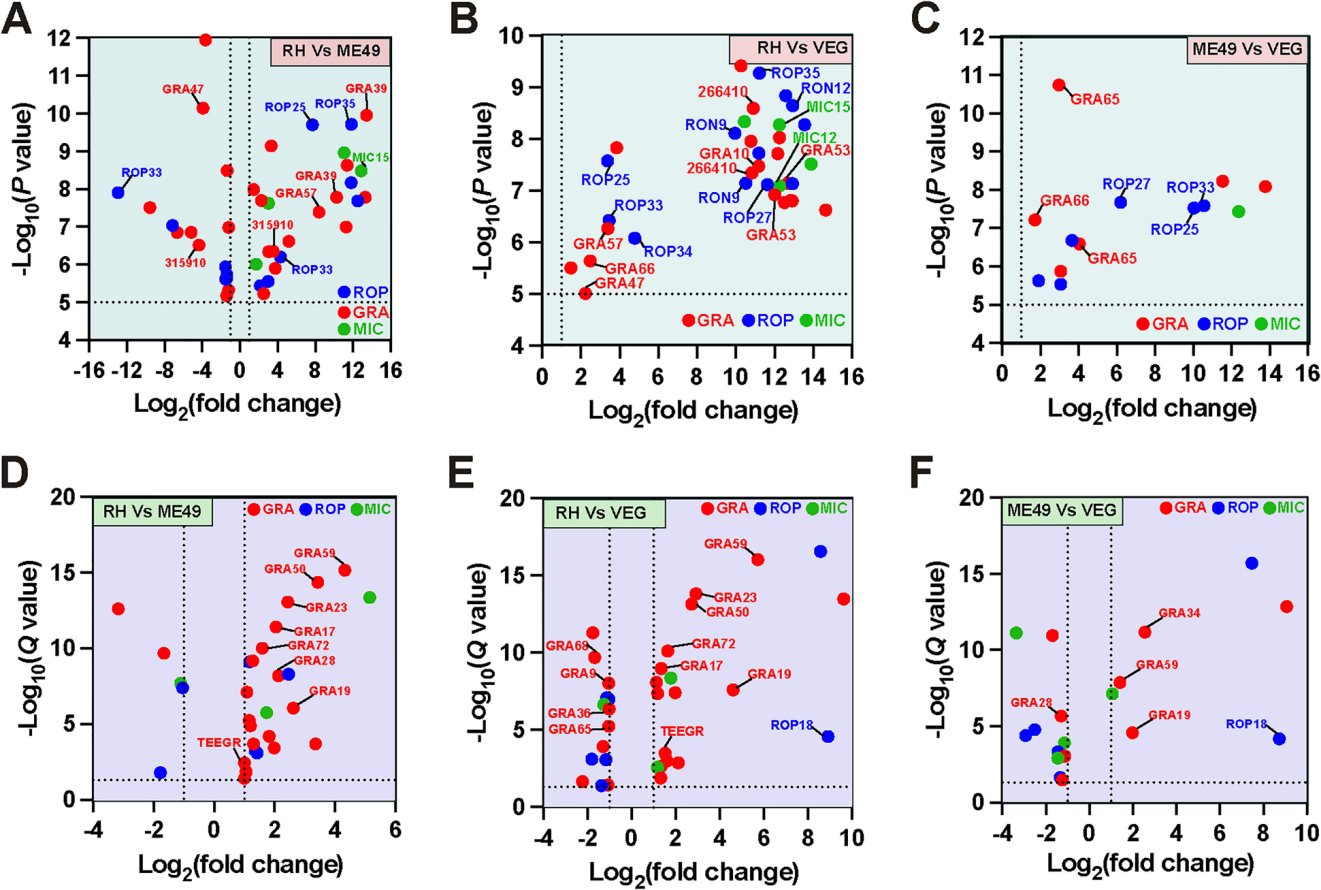
Differentially m^5^C methylated and expressed virulence related effectors, including secreted rhoptry proteins (ROPs), dense granule proteins (GRAs) and microneme proteins (MICs). **A**–**C** Volcano plots displaying differentially methylated effectors in the RH versus ME49, RH versus VEG, and ME49 versus VEG comparisons, respectively. The threshold for significance is indicated by the dotted line corresponding to the *P* value threshold of 10⁻^5^. **D**–**F** Volcano plots displaying differentially expressed effectors in the RH versus ME49, RH versus VEG, and ME49 versus VEG comparisons, respectively. The threshold for significance is indicated by the dotted line corresponding to the *Q* value (adjusted *P*-value) threshold of 0.05. *RH Toxoplasma* wild-type I strain, *ME49 Toxoplasma* wild-type II strain, *VEG Toxoplasma* wild-type III strain

Distinct lineages of *T. gondii* (type I, II and III) exhibit significant differences in virulence [6]. Previous genetic and CRISPR-based screens have identified key virulence-associated effectors, including secreted rhoptry proteins (ROPs), dense granule proteins (GRAs) and microneme proteins (MICs), such as ROP5, ROP18, GRA16 and GRA45 [6, 46–48]. To investigate the m^5^C methylation and expression patterns of those effectors, we focused on differentially methylated or expressed ROPs, GRAs and MICs, based on their predicted localization, as determined by hyperplexed Localization of Organelle Proteins by Isotopic Tagging (hyperLOPIT) (Fig. 8; Additional file 9: Table S8 and Additional file 10: Table S9) [49].

m^5^C methylation analysis revealed hypermethylation of most GRAs, ROPs, and MICs across all three strain comparisons (Fig. 8A–C; Additional file 9: Table S8), although strain-specific virulence factors did not show differential methylation. In the RH versus ME49 comparison, 41 differentially methylated peaks (25 hypermethylated and 16 hypomethylated) were detected across 26 genes, including 12 GRAs, 3 MICs, and 11 ROPs (Fig. 8A). In the RH versus VEG comparison, all 33 differential peaks across 25 secretory effector genes were hypermethylated (Fig. 8B). Similarly, in the ME49 versus VEG comparison, all 11 secretory effector genes showed hypermethylation (Fig. 8C). These differentially methylated genes include known virulence factors, such as ROP35 [50], GRA39 [51], GRA47 [36, 52], TGME49_266410, and TGME49_315910 [53], suggesting a strong role for m^5^C methylation in regulating virulence. Based on our findings, specific candidate genes such as GRA39, ROP35, and GRA47, which have been implicated in *T. gondii* virulence, exhibited robust differential m^5^C methylation patterns that may influence their molecular functions. These findings highlight the potential involvement of m^5^C methylation in regulating key virulence factors.

Transcriptome comparisons of virulence-related genes revealed differential expression of several virulence-related genes. Interestingly, GRA17 [54, 55], GRA23 [54, 55], GRA72 [56, 57] and TEEGR [58] were significantly upregulated in the RH strain compared to ME49 or VEG (Fig. 8D–F, Additional file 10: Table S9). ROP18, which is absent in the type III lineage [59], was upregulated in both the RH versus VEG and ME49 versus VEG comparisons (Fig. 8D–F). Conjoint analysis of methylation and expression data revealed co-varying patterns for genes such as TGME49_208070 (a putative MIC), MIC12, and TGME49_301480 (a putative GRA).

### Expression and m^5^C methylation analysis of potential m^5^C regulators

Dynamic regulatory elements of RNA m^5^C modification have been identified in various species, including methyltransferases (NSUN1–NSUN6, TRM4A, the “writer”), the demethylase ALKBK1 (the “eraser”), and binding proteins such as Aly/REF export factor (ALYREF) and YBX1 (the “readers”) [14, 60]. However, the regulatory landscape of m^5^C modification in *T. gondii* remains largely uncharacterized, with current knowledge mainly focused on DNA methyltransferases [61]. In our analysis, we identified several candidate genes in *T. gondii* homologous to known m^5^C regulators. Many of these genes exhibited negative phenotype scores in genome-wide CRISPR screens, suggesting their essential roles in tachyzoite invasion and proliferation [62].

**Fig. 9:**
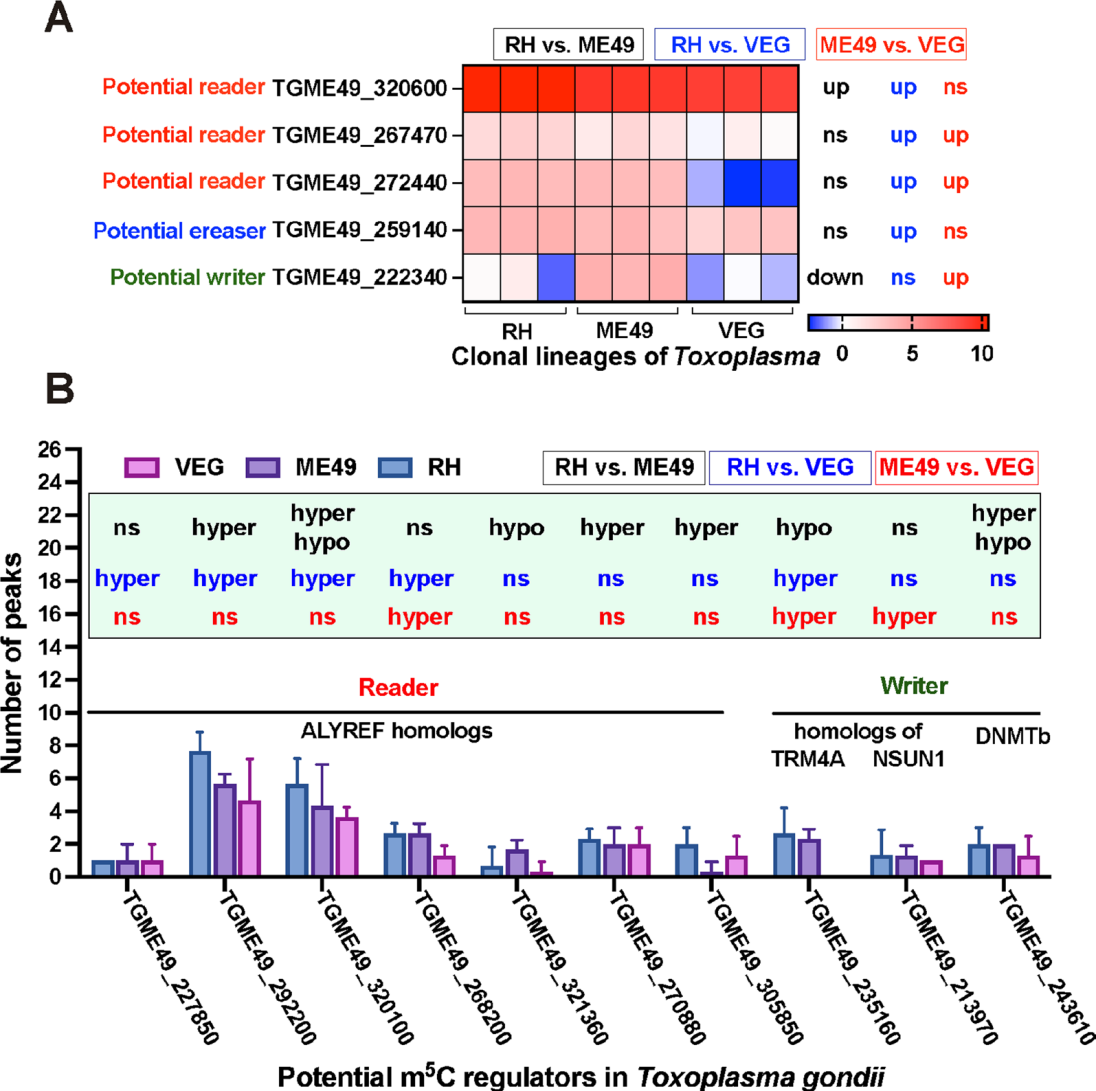
Transcriptional regulation and m^5^C methylation patterns of potential m^5^C regulators. **A** Heatmap showing differential expression of putative m^5^C regulators and pairwise expression comparisons of these potential regulators across *T. gondii* strains. **B** Histogram illustrating the number of m^5^C methylation peaks in differentially methylated m^5^C regulators and pairwise comparisons of these methylation levels in potential regulators across *T. gondii* strains. Statistical significance of expression or methylation in comparison of RH versus ME49, RH versus VEG and ME49 versus VEG were shown in black, blue and red, respectively. *RH Toxoplasma* wild-type I strain, *ME49 Toxoplasma* wild-type II strain, *VEG Toxoplasma* wild-type III strain,* m*^*5*^*C* 5-methylcytosine, *ALYREF* binding protein Aly/REF export factor, *NSUN* NOL1/NOP2/SUN domain family member, *TRM4A* tRNA specific methyltransferase 4A, *DNMTb* DNA methyltransferase b

To investigate the expression patterns of potential m^5^C regulators, we analyzed RNA-Seq data. Two reader homologs (TGME49_267470 and TGME49_272440) were upregulated in the RH and ME49 strains compared to the VEG strain. However, TGME49_320600 (a potential reader) exhibited the highest expression in RH tachyzoites, surpassing both the ME49 and VEG strains (Fig. 9A; Additional file 11: Table S10). An eraser homolog (TGME49_259140) was upregulated in the RH versus VEG comparison, although no significant expression differences were observed in the RH versus ME49, and ME49 versus VEG comparisons (Fig. 9A; Additional file 11: Table S10). Interestingly, a potential m^5^C writer (TGME49_222340) was downregulated in both RH and VEG tachyzoites compared to ME49 tachyzoites.

We next examined the m^5^C methylation status of these candidate regulators to understand the relationship between their expression and methylation patterns. This epitranscriptomic analysis identified 10 potential regulators with differential methylation, including seven homologs of the reader ALYREF (TGME49_227850, TGME49_292200, TGME49_320100, TGME49_268200, TGME49_321360, TGME49_270880, TGME49_305850) and two potential writers (TGME49_235160 and TGME49_213970, homologs of TRM4A and NSUN1, respectively), along with DNMTb (TGME49_243610) (Fig. 9B; Additional file 12: Table S11). Interestingly, multi-peak modifications (i.e., more than one methylation peak per gene) were common among these differentially methylated candidates (Fig. 9B). Two potential readers (TGME49_292200 and TGME49_320100) exhibited more than three methylation peaks in all *T. gondii* strains and were hypermethylated in RH tachyzoites compared to both ME49 and VEG strains (Fig. 9B). Interestingly, bidirectional methylation (i.e., the co-occurrence of hyper- and hypo-methylated sites) was observed in the potential reader (TGME49_320100) and the potential writer (TGME49_243610) during the RH versus ME49 comparison (Fig. 9B). However, no putative regulator exhibited both differential expression and methylation in a coordinated manner, highlighting the complexity of m^5^C regulation.

In addition to the differentially expressed or methylated m^5^C regulatory candidates, we also observed conserved expression and methylation patterns across the three *T. gondii* tachyzoite lineages. This included ALYREF homologs (TGME49_224580, TGME49_318690, TGME49_268200 and TGME49_283740), eukaryotic NSUN homologs (TGME49_294440), and DNMTb homologs (TGME49_227660), all of which exhibited conserved expression patterns (Additional file 11: Table S10). Furthermore, m^5^C-methylated ALYREF homologs (TGME49_294670, TGME49_306600, TGME49_289560, and TGME49_224580) showed no significant difference in m^5^C methylation across the *T. gondii* tachyzoite strains (Additional file 4: Table S3). Interestingly, all these potential regulators had negative knockout fitness scores [62].

## Discussion

RNA modifications, particularly m^5^C methylation, are crucial regulators of gene expression and cellular processes across diverse organisms [11, 14]. While two functional DNA methyltransferases, TgDNMTa and TgDNMTb, have been well-characterized in *T. gondii* [61], the role of m^5^C in regulating the parasite’s pathobiology remains underexplored—particularly in terms of its epitranscriptomic landscape. To address this knowledge gap, we conducted a comprehensive mapping of mRNA m^5^C modifications across three distinct *T. gondii* lineages (RH, ME49, and VEG) using an integrated approach combining m^5^C-MeRIP-Seq and RNA-Seq. Our aim was to unravel the intricate regulatory networks linking methylation to gene expression, with particular focus on its implications for virulence and potential therapeutic targets. The robustness of our findings was validated through both MeRIP-qPCR (for methylation) and RT-qPCR (for expression) assays, ensuring the reliability of our results.

The positional distribution of m^5^C peaks and their associated sequence motifs are key determinants for understanding RNA methylation mechanisms. Our methylome profiling revealed that m^5^C modifications in *T. gondii* tachyzoites are predominantly concentrated in the CDS regions. This pattern mirrors findings in *Arabidopsis thaliana* and *Eimeria tremella* [38, 63] but distinct from that observed in *Oryza sativa* [64]. Interestingly, the most common m^5^C motifs identified in all three *T. gondii* lineages were UGSAK (S = G or C, K = G or U) and GAMGVMG (M = A or C, V = A, C, or G). These motifs are distinct from those reported in *A. thaliana* and *E. tremella*, highlighting both conserved and lineage-specific variations in RNA methylation [38, 63]. This suggests that m^5^C methylation may play a role in the adaptation of *T. gondii* across different species, further emphasizing the diversity of epitranscriptomic regulation in eukaryotes.

Our methylome profiling revealed widespread m^5^C methylation in *T. gondii* tachyzoites, with 59.0–66.2% of total transcripts exhibiting methylation peaks, some containing ≥ 10 methylation sites. Functional annotation of DMMGs across the three *T. gondii* lineages highlighted enrichment in GO terms related to molecular functions such as binding and catalytic activity, cellular components associated with vesicles and membranes, and biological processes including transport, localization, and transcriptional regulation. While this study primarily focused on mRNA m^5^C methylation, it did not explore tRNA and other RNA methylation in detail. Previous research has emphasized the significance of tRNA modifications in *T. gondii* [17], but tRNA m^5^C methylation was outside the scope of the present study. A more in-depth analysis of tRNA m^5^C methylation will be an essential focus in our future work, as it will provide a more comprehensive understanding of the complete landscape of m^5^C regulation across various RNA species.

KEGG pathway analysis further highlighted nucleocytoplasmic transport, DNA replication, and ATP-dependent chromatin remodeling as key pathways regulated by m^5^C-methylation. A comparison with *Plasmodium* revealed a similar methylation-mediated regulation of nucleic acid metabolism, chromatin organization, and nucleoside transport during the asexual stage [32]. Collectively, these findings suggest that m^5^C-mediated epitranscriptomic regulation in *T. gondii* may influence enzyme-driven processes by modulating catalytic domains and chromatin remodelers. This, in turn, could contribute to phenotypic divergence across different *T. gondii* lineages.

Integrated analysis of the epitranscriptome and transcriptome revealed numerous DEGs that co-occurred with differential m^5^C methylation. Interestingly, three HECT domain-containing proteins (TGME49_270580, TGME49_280660, and TGME49_209000), which belong to the E3 ubiquitin-protein ligase family and contain multiple methylation sites, exhibited both differential methylation and expression in the RH versus ME49 comparison. E3 ubiquitin ligases, essential for protein degradation and cellular regulation [65, 66], were found to be influenced by m^5^C methylation. This further links m^5^C modification to crucial processes underlying parasite development and virulence. These findings suggest that phenotypic variations observed among *T. gondii* lineages may be shaped by m^5^C-regulated ubiquitination pathways.

Secretory proteins, including GRAs, ROPs, and MICs, are central virulence factors in *T. gondii*, helping the parasite evade host immunity [6]. The virulence determinant ROP18, highly expressed in type I and II strains but absent in type III, forms a complex with ROP5 to phosphorylate immunity-related GTPase 6 (Irga6). This process, facilitated by GRA7, prevents the binding of immune-related GTPases (IRGs) to the parasitophorous vacuole membrane (PVM), thus avoiding vacuole destruction and promoting tachyzoite survival [67]. ROP35, essential for in vivo cyst burden [50], were hypermethylated in the RH versus ME49 and RH versus VEG comparisons, suggesting m^5^C modification may play a role in chronic infection. Compared to less virulent strains, more virulent strains exhibit hypermethylation in secretory effectors, such as the dense granule proteins GRA39 [51], GRA47 [36, 52], TGME49_266410, and TGME49_315910 [53], suggesting a correlation between m^5^C methylation and increased virulence in *T. gondii*.

Transcriptional analysis further revealed differential expression of known virulence effectors, including ROP18, GRA17, GRA72, and TEEGR, across the strains. Interestingly, while these known virulence factors did not exhibit co-varying patterns of expression and methylation, certain other genes such as TGME49_208070 (a putative MIC), MIC12, and TGME49_301480 (a putative GRA) displayed significant changes in both methylation and expression. These observations prompt further research to determine whether these methylation-expression patterns are directly associated with strain-specific virulence. Based on our findings, we hypothesize that differential m^5^C methylation of key effector genes, such as GRA39 and GRA47, plays a crucial role in regulating their molecular functions (e.g., nutrients uptake) and influencing virulence traits across different *T. gondii* lineages. Functional validation studies, such as gene knockouts or CRISPR-based editing, will be critical to establish a definitive link between m^5^C methylation and pathogenicity.

Our analysis also revealed that AMT2, a gene involved in isoprenoid and fatty acid biosynthesis and known for its role in *T. gondii* virulence in mice [45], was hypomethylated in the RH versus ME49 comparison, despite showing no significant expression changes. In contrast, two major facilitator superfamily (MFS) transporters (TGME49_268020 and TGME49_319740), exhibited a positive correlation between methylation and expression in the ME49 versus VEG comparison. This suggests that m^5^C methylation may play a role in regulating nutrient transport, contributing to strain-specific phenotypic differences. Importantly, the identification of conserved methylated mRNAs across *T. gondii* lineages highlights the potential of targeting these modifications in the development of new anti-parasitic therapeutics.

m^5^C methylation is tightly regulated by a network of “writers,” “readers,” and “erasers” [11, 14, 15]. Over ten RNA m^5^C methyltransferases (writers) have been identified, including members of the NSUN family, tRNA-specific methyltransferases like TRDMT, and DNMTb [11, 14, 15]. Both NSUN2 and TRM4B are known to exhibit mRNA methyltransferase activity across eukaryotes [63, 64, 68, 69]. In apicomplexan parasites, the functions of several m^5^C writers have been characterized, including Pf-NSUN2, Pf-DNMT2, TgDNMTa, and TgDNMTb [32, 33, 61]. PyNSUN2, an ortholog of Pf-NSUN2 in *Plasmodium yoelii*, stabilizes mRNA transcripts and regulates gametocyte development via m^5^C-mediated mechanisms [32]. In our study, TGME49_222340 was differentially expressed, which is a homolog of NSUN1–NSUN6 and TRM4A, identified using BLASTp searches in the ToxoDB database (https://toxodb.org). Given the role of PyNSUN2 in *Plasmodium* gametocyte development, TGME49_222340 may similarly regulate life-cycle transitions in *T. gondii*. Furthermore, three other putative methyltransferases—TGME49_235160 (homologous to TRM4A), TGME49_213970 (NSUN1), and TGME49_243610 (DNMTb)—exhibited differential methylation patterns across *T. gondii* lineages, suggesting their involvement in regulating epitranscriptomic differences and lineage-specific phenotypes.

m^5^C demethylation is facilitated by ten-eleven translocation (TET) family proteins and AlkB homologues (ALKBH) in mammalian cells [70–73]. In *Arabidopsis*, ALKBH6 plays a key role in regulating stress responses by binding to m^5^C-modified RNAs during germination and seedling growth [74]. Through comparative genomic analysis, we identified TGME49_259140 as a homolog of ALKBH6, which exhibited differential expression between the RH and VEG strains. CRISPR screening data from ToxoDB provided a knockout fitness score of − 1.59 for this gene [62], highlighting its potential importance for tachyzoite proliferation. These findings suggest that TGME49_259140 may be involved in epitranscriptomic regulation under stress conditions; however, further experimental validation is required to confirm its role in RNA demethylation.

Readers recognize m^5^C-modified RNA and regulate various downstream processes, including splicing, localization, translation, stability, and degradation [15]. In mammals, the reader protein ALYREF selectively binds m^5^C-methylated mRNAs to facilitate their export [75], while YBX1 plays a role in stabilizing maternal mRNAs and coordinating processes, such as early embryogenesis in zebrafish [76], autophagy [77], and hematopoietic stem cell expansion [78, 79]. In *T. gondii*, we identified two YBX1 homologs (TGME49_320600 and TGME49_267470) and one ALYREF homolog (TGME49_272440), all of which exhibited differential expression across strains. Additionally, seven potential ALYREF-like readers showed strain-specific differences in m^5^C methylation, suggesting that these proteins may interact with m^5^C-modified transcripts and contribute to phenotypic diversity between *T. gondii* strains. Several potential m^5^C regulatory factors exhibited conserved expression or methylation across *T. gondii* tachyzoites, including readers (TGME49_224580, TGME49_318690, TGME49_268200, and TGME49_283740) and writers (TGME49_227660). Interestingly, their negative knockout fitness scores indicate essential roles in *T. gondii* survival, highlighting their potential as therapeutic targets.

While this study provides a comprehensive m^5^C epitranscriptomic landscape in *T. gondii*, several limitations should be noted. First, this study was restricted to mRNA type and the tachyzoite stage. Expanding to other RNA species (e.g., tRNAs, lncRNAs) and other developmental stages (bradyzoites, sporozoites) would offer a more complete understanding of m^5^C dynamics in *T. gondii*. Second, although we identified differentially methylated genes and potential regulators, the functional consequences of these modifications, particularly in virulence-associated mRNAs, require experimental validation through approaches like site-directed mutagenesis or methylation inhibition assays to establish direct mechanistic links. Third, technical limitations in m^5^C detection methods may have affected data sensitivity, suggesting the need for orthogonal validation using emerging technologies like nanopore sequencing. These considerations highlight important directions for future research to fully elucidate the regulatory roles of m^5^C in parasite biology.

## Conclusions

Our study provides a comprehensive characterization of the m^5^C epitranscriptome in *T. gondii* tachyzoites of the three major clonal lineages, uncovering both conserved and strain-specific methylation patterns that may contribute to strain-specific phenotypic variations. Through integrated MeRIP-Seq and RNA-Seq analyses, we identified differentially methylated and expressed genes that likely play pivotal roles in parasite virulence and adaptation. Our findings suggest that lineage-specific m^5^C regulators may drive differential pathogenicity, while conserved regulatory factors may be essential for *T. gondii* infection, positioning them as potential targets for vaccine development or therapeutic intervention. Future research should focus on functional validation of candidate regulators using CRISPR-Cas9 technology and site-directed mutagenesis of conserved m^5^C sites in virulence-related mRNAs to establish direct links between methylation and pathogenicity.

## Supplementary Information

Additional file 1 (Table S1. Primer sequences for RT-qPCR analysis.)

Additional file 2 (Table S2. Primer sequences for MeRIP-qPCR analysis.)

Additional file 3 (Figure S1. Distribution of m^5^C methylation peaks across chromosomes and gene percentages with varying numbers of m^5^C methylation sites in *T. gondii* tachyzoites. Figure S2. Chromosomal distribution of differential m^5^C methylation peaks and gene counts with differential methylation in *T. gondii* tachyzoites. Figure S3. Volcano plots of differentially expressed genes. Figure S4. Correlation analysis between differential mRNA expression levels and differential m^5^C methylated levels.).

Additional file 4 (Table S3. Differentially methylated mRNA sites.)

Additional file 5 (Table S4. Differentially enriched GO terms associated with differentially methylated mRNA peaks.)

Additional file 6 (Table S5. KEGG pathway terms for mRNA with differentially methylated peaks.)

Additional file 7 (Table S6. Differentially expressed genes.)

Additional file 8 (Table S7. Conjoint analysis of differentially expressed mRNAs and methylation peaks.)

Additional file 9 (Table S8. Differentially methylated ROPs, GRAs and MICs.)

Additional file 10 (Table S9. Differentially expressed ROPs, GRAs and MICs.)

Additional file 11 (Table S10. Expression patterns of potential m^5^C regulators.)

Additional file 12 (Table S11. Differential m^5^C methylation of potential m^5^C regulators.)

## Data Availability

The datasets supporting the findings of this article are provided within the paper and supplementary materials. The raw sequence data have been deposited in the NCBI Gene Expression Omnibus repository under accession number GSE294543.

## Abbreviations

m^5^C: 5-Methylcytosine
mRNA: Messenger RNA
MeRIP-Seq: Methylated RNA immunoprecipitation sequencing
CDS: Coding sequence
DMMGs: Deferentially m^5^C methylated genes
DEGs: Differentially expressed genes
RFLP: Restriction fragment length polymorphism
m^6^A: N6-methyladenosine
m^1^A: N1-methyladenosine
m^7^G: N7-methylguanosine
NSUN: NOL1/NOP2/SUN domain family member
NSUN2: NOL1/NOP2/SUN domain family member 2
YBX1: Reader protein Y-box binding protein 1
HFF cells: Human foreskin fibroblast cells
DMEM: Dulbecco’s Modified Eagle Medium
FBS: Fetal bovine serum
m^5^C-MeRIP-Seq: M^5^C methylated RNA immunoprecipitation sequencing
IP: Immunoprecipitation
FC: Fold change
GO: Gene Ontolog
KEGG: Kyoto Encyclopedia of Genes and Genomes
RT-qPCR: Quantitative reverse transcription PCR
UTR: Untranslated region
MF: Molecular functions
CC: Cellular components
BP: Biological processes
ROP: Rhoptry protein
GRA: Dense granule protein
MIC: Microneme protein
AMT2: Apicomplexan monocarboxylate transporter 2
ALYREF: Binding protein Aly/REF export factor
ALKBH: AlkB homologues
TET: Ten-eleven translocation family proteins
DNMT: DNA methyltransferase
TRDMT: TRNA specific methyltransferase family member
