# Supplementary material for: Cross-lineage 5-methylcytosine methylome profiling reveals methylated divergence among Toxoplasma gondii tachyzoites of the three major clonal lineages
Source: Infect Dis Poverty. 2025 Aug 19;14:87. doi: 10.1186/s40249-025-01358-w (PMC12362873; doi:10.1186/s40249-025-01358-w)
Supplement: Supplementary file 3 — Additional file 3 (Figure S1. Distribution of m5C methylation peaks across chromosomes and gene percentages with varying numbers of m5C methylation sites in T. gondii tachyzoites. Figure S2. Chromosomal distribution of differential m5C methylation peaks and gene counts with differential methylation in T. gondii tachyzoites. Figure S3. Volcano plots of differentially expressed genes. Figure S4. Correlation analysis between differential mRNA expression levels and differential m5C methylated levels.). [file 40249_2025_1358_MOESM3_ESM.pdf]

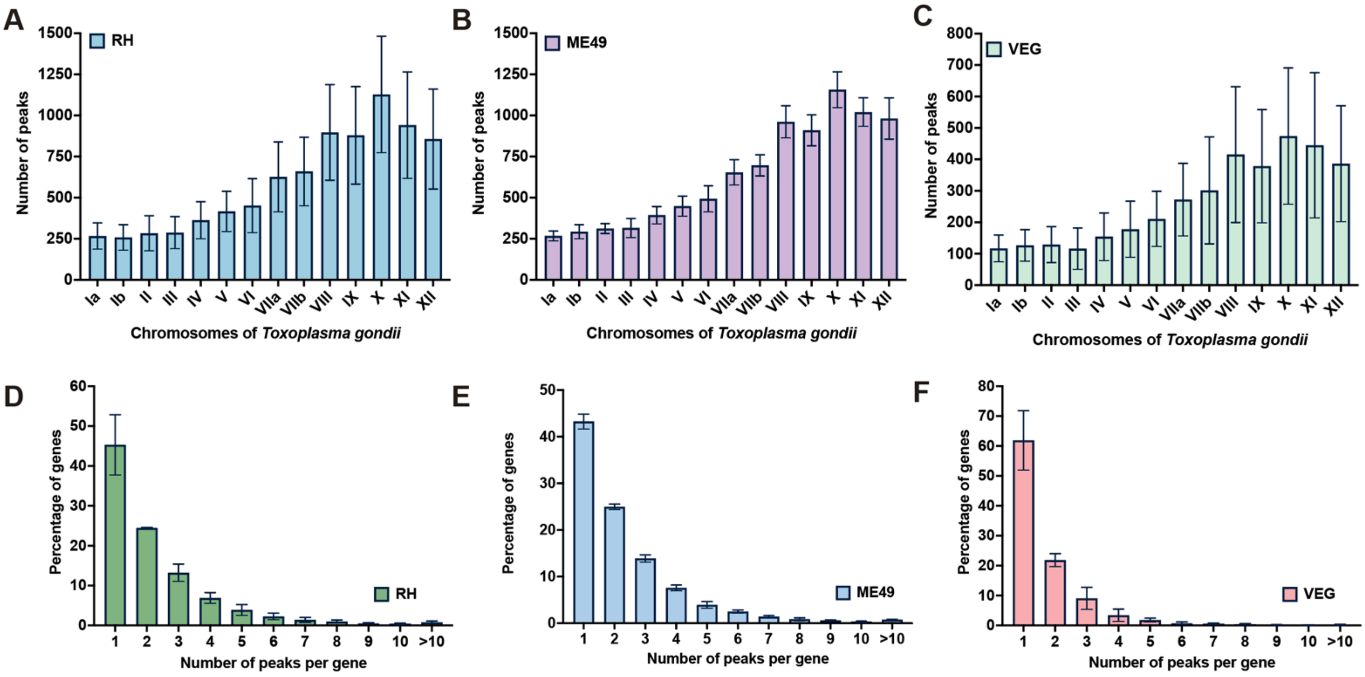

Additional file 3: Figure S1. Distribution of 5-methylcytosine (m^5C) methylation peaks across chromosomes and gene percentages with varying numbers of m^5C methylation sites in *T. gondii* tachyzoites. (**A**–**C**) Chromosomal distribution of m^5C methylation peaks in the RH, ME49, and VEG strains. (**D**–**F**) Proportions of genes with different numbers of m^5C methylation peaks in the RH, ME49, and VEG strains. *RH Toxoplasma* wild-type I strain, *ME49 Toxoplasma* wild-type II strain, *VEG Toxoplasma* wild-type III strain.

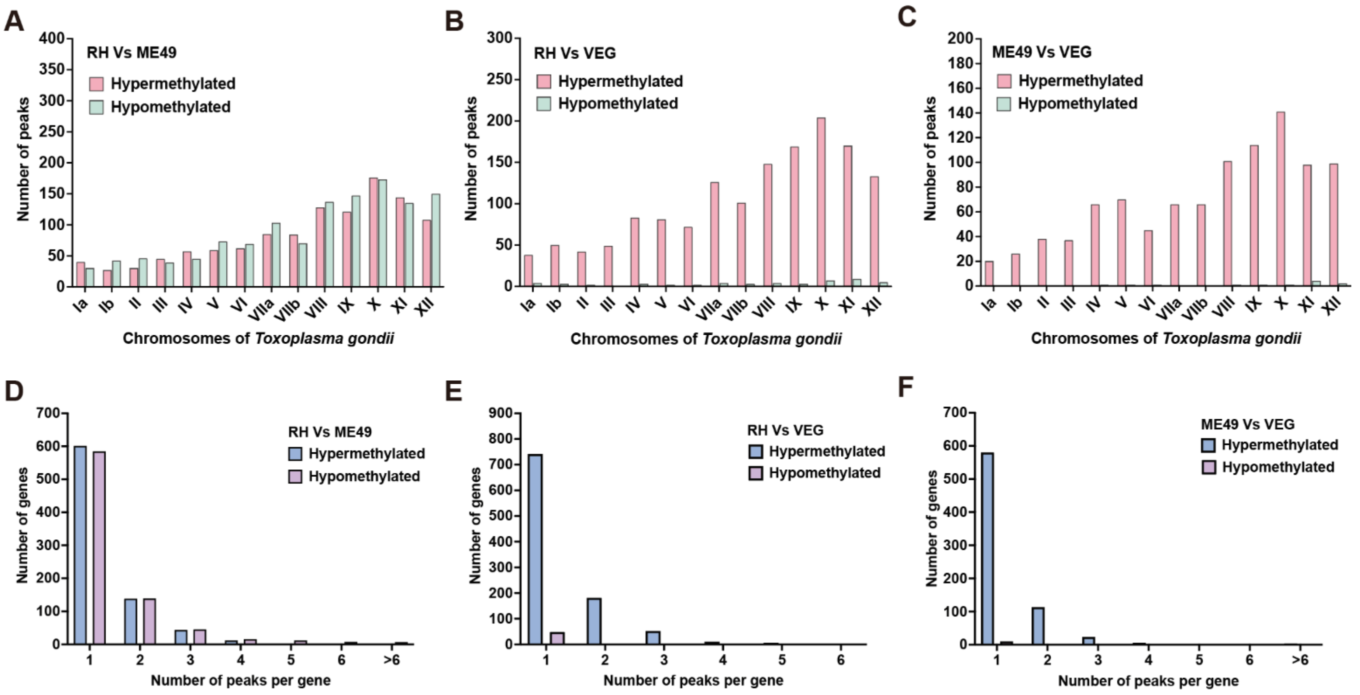

Additional file 3: Figure S2. Chromosomal distribution of differential 5-methylcytosine (m^5C) methylation peaks and gene counts with differential methylation in *T. gondii* tachyzoites. (**A**–**C**) Distribution of hypermethylated and hypomethylated peaks across chromosomes in RH vs. ME49, RH vs. VEG, and ME49 vs. VEG comparisons. (**D**–**F**) Number of genes with differentially methylated peaks across the same comparisons. *RH Toxoplasma* wild-type I strain, *ME49 Toxoplasma* wild-type II strain, *VEG Toxoplasma* wild-type III strain.

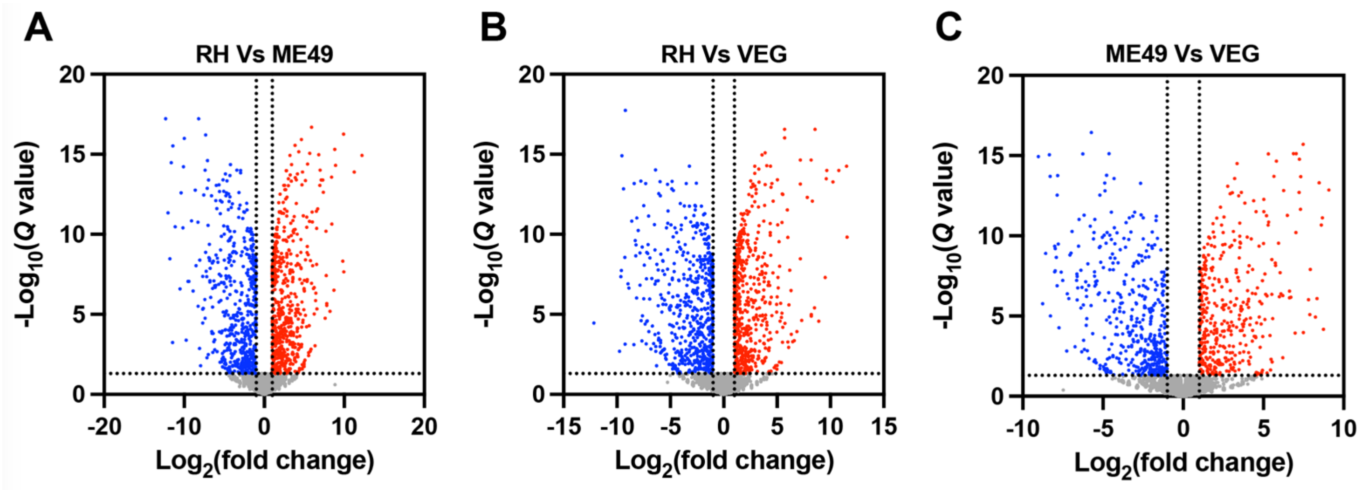

Additional file 3: Figure S3. Volcano plots of differentially expressed genes. Volcano plots showing differentially expressed mRNAs in RH vs. ME49 (**A**), RH vs. VEG (**B**), and ME49 vs. VEG (**C**) comparisons. The plots illustrate the relationship between the fold change (x-axis) and statistical significance (y-axis) for each gene. Genes with significant differential expression are highlighted, with upregulated genes in red and downregulated genes in blue. The threshold for significance is indicated by the dotted line corresponding to the *Q* value (adjusted *P*-value) threshold of 0.05. *RH Toxoplasma* wild-type I strain, *ME49 Toxoplasma* wild-type II strain, *VEG Toxoplasma* wild-type III strain.

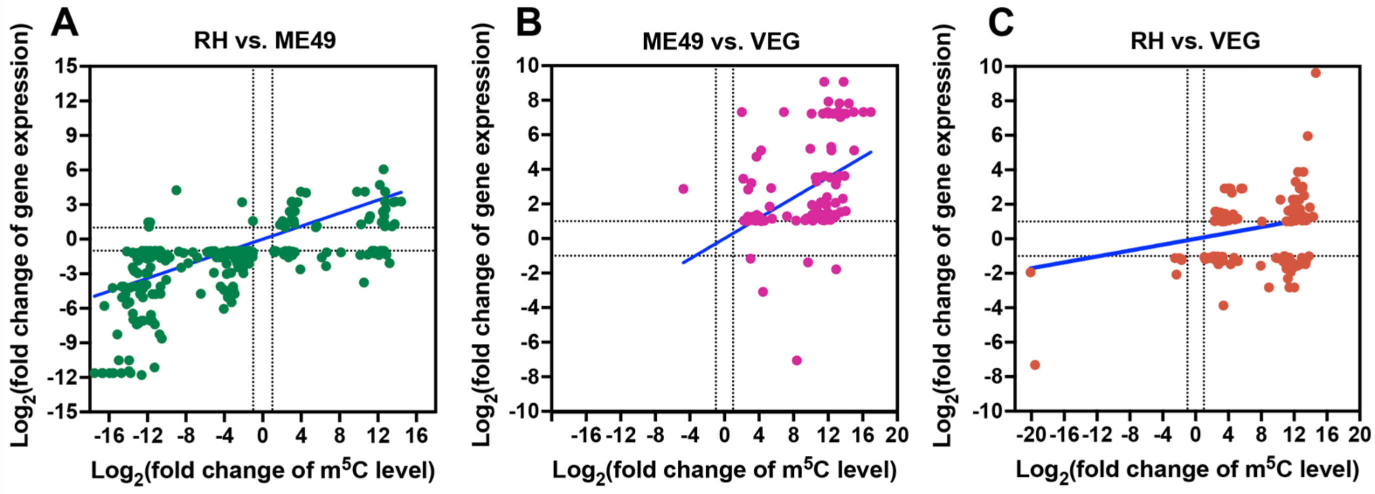

Additional file 3: Figure S4. Correlation analysis between differential mRNA expression levels and differential m^{5}C methylated levels in RH vs. ME49 (**A**), RH vs. VEG (**B**), and ME49 vs. VEG (**C**) comparisons. *RH Toxoplasma* wild-type I strain, *ME49 Toxoplasma* wild-type II strain, *VEG Toxoplasma* wild-type III strain.
